# Exploring relationships between self-compassion, impostor phenomenon, and mental health among doctoral students

**DOI:** 10.3389/fpsyg.2025.1669075

**Published:** 2025-11-25

**Authors:** Brian J. Clarke, Michael T. Hartley

**Affiliations:** Department of Disability and Psychoeducational Studies, The University of Arizona, Tucson, AZ, United States

**Keywords:** impostor phenomenon, self-compassion, mental health, graduate education, doctoral students

## Abstract

**Introduction:**

Impostor phenomenon (IP) is widespread in doctoral education and is strongly linked to mental distress. Self-compassion is theoretically and empirically positioned as a counterbalance to the harsh self-evaluation embedded in IP, yet large-scale evidence in doctoral populations is limited.

**Methods:**

A national sample of 1,225 U.S. doctoral students completed validated measures of IP, self-compassion, anxiety, depression, and loneliness. Data were collected via online survey. Analyses included descriptive statistics, correlations, MANOVA tests comparing mental health across IP tertiles, and three hierarchical regression models testing whether self-compassion explained additional variance in distress beyond IP.

**Results:**

IP scores were skewed high; women and non-binary students reported the highest IP. Anxiety, depression, and loneliness rose as IP increased. Across all three outcomes, self-compassion explained substantial additional variance beyond IP (ΔR² = 0.08–0.10) and meaningfully reduced IP’s standardized coefficients (47%–75%). When self-compassion was added, IP was no longer associated with loneliness, and its associations with depression and anxiety were weakened. MANOVA showed large-effect, stepwise increases in distress from low to moderate IP, while self-compassion declined at each IP level.

**Discussion:**

IP was widespread in this sample and was associated with substantially elevated levels of distress, including at low to moderate IP levels. Self-compassion demonstrated robust inverse associations with loneliness, anxiety, and depression even when IP was considered in the models. The pattern of results indicates that self-compassion is a salient correlate of mental health in the context of IP among doctoral students.

## Introduction

Doctoral students often experience impostor feelings, such as doubting their abilities, feeling inadequate, and attributing their achievements to luck rather than their own ability (Pervez et al., 2020; Sverdlik et al., 2020; Wang and Li, 2023). Clance and Imes (1978) first described the Impostor Phenomenon (IP) as an internal experience of intellectual phoniness despite success and accolades such as degrees, awards, promotions, and even tenure. Building on the initial work of Clance and Imes, researchers have found impostor feelings to be prevalent among a wide range of high-achieving people, especially ethnic minority groups, women, and non-binary individuals (Bravata et al., 2020; Patzak et al., 2017; Rackley et al., 2024).

Rooted in unrealistic expectations and perfectionism, IP is the experience of feeling like a fraud despite a record of success (Garba et al., 2024; Posselt, 2018). IP does not occur in a contextual vacuum, and the culture of higher education can exacerbate impostor feelings in doctoral students (Cohen and McConnell, 2019; Parkman, 2016). Without emotional support and regulating strategies like self-compassion, doctoral education can be an isolating experience characterized by pervasive feelings of inadequacy and self-doubt (Bano and O'Shea, 2023; Jackman et al., 2022).

Research indicates that impostor feelings are common among doctoral students. In a systematic review, Bravata et al. (2020) found that a significant portion (over 50%) of doctoral students reported feeling not qualified or competent despite their high academic achievements. In another review, Wang and Li (2023) found that 50–75% of doctoral students experienced elevated IP (Pervez et al., 2020; Lee et al., 2020). In applied graduate degrees like psychology and counseling, Clarke et al. (2024) noted that 65.1% of students experienced elevated IP, a finding that supported Roskowski (2010) and Tigranyan et al. (2021). These studies not only suggest that IP is common but also provided important insights into the influence of IP on anxiety, depression, and diminished self-efficacy.

Impostor feelings contribute to the mental health crisis among graduate students (Bravata et al., 2020; Evans et al., 2018; Mills et al., 2024). Graduate students are at least six times more likely than the general population to suffer from depression and anxiety (Evans et al., 2018). Connected with IP, persistent self-doubt and school-related stressors are among the most significant contributors to mental distress among doctoral students (Evans et al., 2018; Solms et al., 2024). The emotional impact of IP stemming from constant hypervigilance and fear of being exposed as a ‘fraud’ can lead to serious mental distress and diminished performance (Blondeau, 2024; Garba et al., 2024). Numerous studies have reported strong relationships between IP and depression, anxiety, and loneliness (Clarke et al., 2024; Bravata et al., 2020; Pervez et al., 2020). If left unchecked, IP often leads to burnout and mental health challenges, including suicidal ideation, which can have a detrimental impact on success in both academic and professional environments (Blondeau, 2024; Garba et al., 2024).

Rather than viewing IP as a personality trait linked with pathology, scholars have argued that contextual factors may cause or trigger IP among doctoral students (Cohen and McConnell, 2019; Parkman, 2016). In doctoral education, high-achieving students are expected to excel and compete for academic prestige and resources with other high-achieving students (Bano and O'Shea, 2023). The pressure to excel can contribute to impostor feelings without faculty mentorship and peer support to help clarify what success looks like in academia (Pervez et al., 2020; Sverdlik et al., 2020; Wang and Li, 2023).

Support and mentorship are essential as doctoral students often experience high-stakes assessment and evaluation, rejection of proposals and manuscripts, and failed experiments that can trigger IP (Parkman, 2016; Posselt, 2018). IP goes well beyond occasional self-doubt or inadequacy and functions as a harmful self-sustaining cycle reinforced by cognitive distortions and misattributions (Clance, 1985; Clance and Lawry, 2024). As part of the impostor cycle, those with IP are more likely to internalize mistakes and setbacks as definitive evidence of their perceived incompetence (Gadsby and Hohwy, 2024). After experiencing success, the relief is short-lived and quickly replaced by the anxiety of replicating the accomplishment with even higher expectations (Clance and Lawry, 2024).

Impostor phenomenon can hinder the internalization of success as a personal achievement, often leading to maladaptive coping, such as perfectionism or procrastination stemming from anticipatory failure (Clance and Lawry, 2024; Gadsby and Hohwy, 2024). Rooted in inaccurate self-evaluations, IP can hinder academic growth by fostering harmful comparisons to unrealistic standards and preventing students from distinguishing their strengths from areas for further development (Pervez et al., 2020; Rosenscruggs and Schram, 2024). The fear and shame associated with IP often mean people suffer in isolation rather than sharing their concerns (Lane, 2015; Gadsby and Hohwy, 2024). IP can be a lonely pattern of negatively skewed self-evaluation and perceived inadequacy that can limit development by making it hard for doctoral students to celebrate their strengths and approach areas they need to actually improve on (Rosenscruggs and Schram, 2024). Self-compassion is a useful framework for understanding how IP relates to negative mental health outcomes.

Self-compassion and IP have an inverse theoretical connection (Clarke et al., 2024; Liu et al., 2023; Patzak et al., 2017). In contrast to the harsh self-criticism of IP, self-compassion is an active process of accepting mistakes and normalizing failures while simultaneously acknowledging one’s capability and worth (Neff, 2023). At its core, self-compassion involves treating oneself with the same kindness one would offer to a loved one during difficult times (Neff, 2023). It includes three main components: self-kindness, common humanity, and mindful awareness. Self-kindness encourages gentleness over self-criticism, while common humanity reminds individuals that setbacks are part of a shared human experience, promoting connection rather than isolation. Mindful awareness allows individuals to acknowledge painful thoughts and feelings without avoidance or becoming overwhelmed by them, leading to a balanced approach to self-assessment (Neff et al., 2005).

Self-compassion is associated with positive mental health, resilience, and decreased self-criticism and shame (Clarke and Hartley, 2024; Neff, 2023). A recent study of graduate students found that self-compassion reduced the negative impact of IP on professional development and was related to improved mental health (Clarke et al., 2024). This is because self-compassion is not dependent on achievement or success, but rather, it is the process of easing the fear of failure and enhancing emotional presence regardless of current performance (Gadsby and Hohwy, 2024).

As a potential antidote to the self-doubt associated with negative self-assessments, self-compassion is associated with lower mental distress due to IP and may help doctoral students navigate the challenges of doctoral education more effectively (Patzak et al., 2017; Richardson et al., 2020). With strong theoretical links between IP, self-compassion, and mental health, there is a need for more research on the utility of a self-compassion framework to counteract the mental distress associated with IP among doctoral students (Patzak et al., 2017; Liu et al., 2023).

Three research questions guided the study: (1) Is IP common among doctoral students in this sample, and does it vary significantly across gender, race or ethnicity, age, field of study, and matriculation status? (2) What are the relationships between IP, anxiety, depression, and loneliness and are there a differential associations related to IP level? (3) To what extent does self-compassion account for variance in loneliness, anxiety, and depression beyond that explained by IP? Informed by previous research, we anticipated that IP scores would be skewed to the high end of the scale and that IP would have a significant negative relationship with anxiety, depression, and loneliness. Finally, we anticipated that self-compassion would be positively associated with mental health, beyond variance explained by IP (Clarke et al., 2024). In other words, when considering self-compassion, the negative relationship between IP and mental health would be significantly reduced.

## Methods

### Procedures

After approval from an Institutional Review Board, data collection occurred via an online Qualtrics survey that participants completed between May 8 and June 5, 2023. Using public listservs on university websites, recruitment emails were sent to doctoral students enrolled at universities across the U. S. The recruitment email described the study, inclusion criteria, and informed consent. Eligibility to participate in the study required individuals to be over the age of 18 and currently enrolled in a doctoral program in the U. S. Once doctoral students consented to participate, they were given access to the online survey which contained the measures described below and a demographic survey written by the authors. Participants concluded their involvement in the study by completing the online survey or withdrawing voluntarily.

### Participants

The sample of 1,225 doctoral students represented universities from all 50 states in the U. S. The average age was 30.3 (*SD* = 6.75) with a range from 21 to 73, and a median age of 27. Most participants self-identified as White (*n* = 719, 58.7%). Smaller percentages identified as Asian (*n* = 166, 13.5%), Hispanic or Latinx (*n* = 106, 8.7%), African American or Black (*n* = 44, 3.6%), multiracial (*n* = 36, 2.9%), Middle Eastern (*n* = 21, 1.7%), American Indian or Alaskan Native (*n* = 8, 0.7%), and Native Hawaiian (*n* = 2, 0.2%). Some of the participants chose not to identify their race/ethnicity (10.0%, *n* = 123). Of the 1,225 participants, 702 (57.3%) identified as women, 338 (27.6%) as men, and 71 (5.8%) as non-binary. Some participants (*n* = 114, 9.3%) chose not to disclose their gender identity.

The academic disciplines of participants included the social sciences (*n* = 489, 39.9%), natural sciences (*n* = 281, 23.0%), humanities (*n* = 131, 10.8%), business and public policy (*n* = 97, 7.9%), education (*n* = 33, 2.7%), health fields (*n* = 32, 2.6%), engineering (*n* = 31, 2.5%), law (*n* = 21, 1.7%), Some participants chose not to report their discipline (*n* = 110, 8.9%). In terms of matriculation, the sample consisted of 213 (17.4%) first year, 155 (12.7%) second year, 204 (16.6%) third year, 195 (15.9%) fourth year, 358 (29.2%), fifth year or more, and 100 (8.2%) chose not to disclose their year of study.

### Measures

#### Clance impostor phenomenon scale (CIPS-10)

The CIPS-10 (Wang et al., 2022) is a 10-item, brief version of the original 20-item CIPS to measured impostor feelings (Clance, 1985). The CIPS-20 is a well-supported as a reliable and valid measure of IP (Mak et al., 2019). Wang et al. (2022) reported the CIPS-10 scores showed good internal reliability (*α* = 0.93) with an overall total score that related strongly to the 20-item total scores using the same data. A recent factorial analysis identified a bi-factor structure that included a general IP factor and three subscale factors *luck, fear of failure*, and *discount* (Brauer and Proyer, 2025). The CIPS-10 employs a 5-point Likert response ranging from 1 (not at all true) to 5 (very true). Items included “I’m afraid people important to me may find out that I’m not as capable as they think I am” and “I feel my success was due to some kind of luck rather than competence.” In the present sample, CIPS-10 scores showed good internal consistency (*α* = 0.88).

Because no validated cutoff scores exist for the CIPS-10, participants were divided into tertile groups based on sample distribution: low (≤33rd percentile), moderate (34th–66th percentile), and high (≥67th percentile).

#### Self-compassion scale-short form (SCS-SF)

The SCS-SF was used to assess self-compassion. This scale is a shortened version of the Self-Compassion Scale (Neff, 2003), consisting of 12 items selected from the original SCS (Raes et al., 2011). Respondents rate each item on a 5-point Likert scale, ranging from 1 (almost never) to 5 (almost always). Example items include “I try to see my failings as part of the human condition” and “I’m disapproving and judgmental about my own flaws and inadequacies” (Neff, 2003, p. 2). The SCS-SF has shown strong internal consistency (*α* = 0.86) and its total score correlates almost perfectly (*r* = 0.98) with the full SCS (Raes et al., 2011). Factor analysis further confirmed that the SCS-SF retained the same factor structure as the original scale (Neff et al., 2019; Raes et al., 2011). SCS-SF mean scores are interpreted as follows: (a) low (1–2.4), (b) moderate (2.5–3.5), and (c) high (3.51–5). In the current study, the SCS-SF demonstrated good internal consistency (*α* = 0.88).

#### Patient health questionnaire-4 (PHQ-4)

The Patient Health Questionnaire-4 (PHQ-4) is a brief, four-item self-report screening instrument designed to assess symptoms of depression and anxiety (Kroenke et al., 2009). Responses are measured on a Likert scale ranging from 0 (“not at all”) to 3 (“nearly every day”), with intermediate options including 1 (“several days”) and 2 (“more than half the days”). Recent findings have demonstrated that the PHQ-4 possesses strong internal consistency (*α* = 0.89) and produces valid, diagnostically reliable measures of depression and anxiety (Khubchandani et al., 2016). Currently, PHQ-4 scores demonstrated good internal consistency (*α* = 0.87).

#### 3-item loneliness scale

The 3-Item Loneliness Scale is a shortened version of the Revised UCLA Loneliness Scale (Hughes et al., 2004). Respondents rate each item on a Likert-type scale from 1 (hardly ever) to 3 (often), with the total score ranging from 3 to 9, where higher scores indicate greater loneliness. The items assess how often individuals felt a lack of companionship, left out, or isolated from others. This brief scale has demonstrated satisfactory reliability, as well as concurrent and discriminant validity, in large U. S. samples (Hughes et al., 2004). In the current study, the scale showed acceptable internal consistency (*α* = 0.82).

### Data analysis

All analyses were conducted using SPSS (v30) with a conservative benchmark (*p* < 0.001) to reduce the likelihood of Type I error and emphasize practically meaningful effects. Descriptive statistics and bivariate correlations explored the relationships between IP, anxiety, depression, loneliness, and self-compassion. The authors screened the data and found it met the criteria for regression analysis and MANOVA including verifying the data was normally distributed, homoscedastic, without outliers, and that the relationships among the variables were linear. Multicollinearity was not evident as all VIF scores were < 2. Three hierarchical regression analyses were used to determine if self-compassion explains variance in loneliness, anxiety, and depression beyond what is explained by IP.

MANOVA was used to test if self-compassion, anxiety, depression, and loneliness vary across levels of IP. Because the CIPS-10 does not have validated cutoff scores, IP scores were divided into three groups (low, moderate, and high) based on sample-specific tertile splits (33rd and 66th percentiles) of total IP scores. This percentile approach provided an empirically grounded and replicable method for examining relative levels of IP within the sample while maintaining approximately balanced group sizes for multivariate analysis. Grouping scores in this way allowed for a meaningful comparison of self-compassion, anxiety, depression, and loneliness across varying degrees of IP intensity. In the MANOVA analysis, partial eta squared (*η*_p_^2^) values determined effect sizes using the convention range for small (*η*_p_^2^ > 0.01), medium (*η*_p_^2^ > 0.059), and large (*η*_p_^2^ > 0.14) effects (Richardson, 2011). Effect sizes for MANOVA follow-up analysis were interpreted according to the general guideline for Cohen’s *d*, small (*d* ≥ 0.2), medium (*d* ≥ 0.5), and large effect sizes (*d* ≥ 0.8; Gignac and Szodorai, 2016).

## Results

### Descriptive statistics

The results revealed impostor phenomenon (IP) and mental distress mean scores were skewed to the higher end of the respective scales. The mean score on the CIPS-10 (*M* = 3.43, *SD* = 0.86) were consistent with recent samples of graduate students (Clarke et al., 2024; Sverdlik et al., 2020). MANOVA revealed that the IP scores did not differ based upon race (*p* = 0.41), area of study (*p* = 0.34), or matriculation (*p* = 0.49). However, IP did vary across age *F*(2, 1,114) = 9.694, *p* < 0.001, Wilk’s *Λ* = 0.995, *η*_p_^2^ = 0.017, with a small effect size. Higher IP scores were reported among participants aged 20–29 (*M* = 3.57 *d* = 0.428, *p* < 0.001) and 30–39 (*M* = 3.37 *d* = 0.245, *p* < 0.001) compared to participants aged 40 or older (*M* = 3.16). IP scores also differed by gender *F*(2, 1,108) = 15.892, *p* < 0.001; Wilk’s *Λ* = 0.946, *η*_p_^2^ = 0.028 with a small effect size. Those who identified as women (*M* = 3.55, *d* = −0.353, *p* < 0.001) and non-binary (*M* = 3.63, *d* = −0.455, *p* < 0.001) had higher IP scores than those who identified as men (*M* = 3.23).

The bivariate correlations revealed statistically significant relationships between IP, loneliness, depression, anxiety, and self-compassion (Table 1). Mental distress scores were elevated with mean scores for loneliness (*M* = 6.23; SD = 1.93), depression (*M* = 2.09; *SD* = 1.92), and anxiety (*M* = 3.20; *SD* = 1.96). Loneliness, depression, and anxiety did not differ across race, age, discipline, and matriculation. Compared to male students, anxiety was significantly higher *F*(2, 1,108) = 9.151, *p* < 0.001, Wilk’s *Λ* = 0.946, *η*_p_^2^ = 0.027 in both female (*M* = 3.36 *d* = −0.274, *p* < 0.001) and non-binary (*M* = 3.41 *d* = −0.302, *p* = 0.01) participants, with a small effect size. The self-compassion mean (*M* = 2.81; *SD* = 0.75) fell in the low-moderate range (Raes et al., 2011), and did not differ based on race, gender, age, or matriculation.

**Table 1:** Descriptive statistics and bivariate correlations.

	*M*	*SD*	1	2	3	4	5
1. Self-Compassion	2.81	0.75	–				
2. Loneliness	6.23	1.93	−0.42*	–			
3. Depression	2.09	1.92	−0.46*	0.47*	–		
4. Anxiety	3.20	1.96	−0.53*	0.37*	0.59*	–	
5. Impostor	3.43	0.86	−0.58*	0.29*	0.39*	0.47*	–

*p ≤ 0.001.

### Hierarchical regression analyses

Three hierarchical multiple regression analyses were conducted to examine whether self-compassion explained variance in loneliness, anxiety, and depression beyond that accounted for by impostor phenomenon (IP). In each model, IP was entered in Step 1, followed by self-compassion in Step 2.

The first regression model examined loneliness as the dependent variable (see Table 2). In Step 1, IP was significantly associated with loneliness, *R*^2^ = 0.083, *F*(1, 1,206) = 108.47, *p* < 0.001, accounting for 8.3% of the variance. Higher levels of IP were associated with greater loneliness (*β* = 0.287, *p* < 0.001). In Step 2, self-compassion significantly improved the model, *R*^2^ = 0.180, *F*(2, 1,205) = 132.61, *p* < 0.001. This model explained 18.0% of the variance in loneliness, representing a significant increase of 9.8%, Δ*R*^2^ = 0.098, *F*(1, 1,205) = 143.90, *p* < 0.001. When considering self-compassion in the model, the relationship between IP and loneliness was non-significance (*β* = 0.071, *p* = 0.025). Self-compassion demonstrated a strong negative association with loneliness (*β* = −0.380, *p* < 0.001). The effect size for self-compassion was large (*f*^2^ = 0.30).

**Table 2:** Examining variance in loneliness explained by IP and self-compassion.

	*B*	*SE*	*β*	*t*	*p*	95% CI
Step 1						
Constant	4.02	0.22		18.42	<0.001	[3.60, 4.59]
Impostor	0.64	0.06	0.29*	10.42	<0.001	[0.52, 0.76]
Step 2						
Constant	8.45	0.42		19.98	< 0.001	[7.62, 9.28]
Impostor	0.16	0.07	0.07	2.24	0.025	[0.02, 0.30]
Self-compassion	−0.98	0.08	−0.38*	−11.99	< 0.001	[−1.14, −0.82]

*p ≤ 0.001.

The second hierarchical multiple regression (Table 3) was conducted to determine if self-compassion explained significant variance in anxiety beyond what IP accounted for in the model. In step 1, IP was significantly related to anxiety, *R*^2^ = 0.218, *F*(1, 1,206) = 336.03, *p* < 0.001, explaining 21.8% of the variance. Higher IP was associated with elevated anxiety (*β* = 0.467, *p* < 0.001). The addition of self-compassion in Step 2 strengthened the model, *R*^2^ = 0.320, *F*(2, 1,205) = 283.34, *p* < 0.001, accounting for 32.0% of the variance in anxiety. This represented a significant increase of 10.2%, Δ*R*^2^ = 0.102, *F*(1, 1,205) = 180.61, *p* < 0.001. When self-compassion was included, the association between IP and anxiety remained significant but was substantially weaker (*β* = 0.246, *p* < 0.001), while self-compassion was associated with lower anxiety (*β* = −0.388, *p* < 0.001). The effect size for self-compassion was large (*f*^2^ = 0.35).

**Table 3:** Examining variance in anxiety explained by IP and self-compassion.

	*B*	*SE*	*β*	*t*	*p*	95% CI
Step 1						
Constant	−0.46	0.21		−2.22	0.027	[−0.87, −0.05]
Impostor	1.07	0.06	0.47*	18.33	< 0.001	[0.95, 1.18]
Step 2						
Constant	4.15	0.39		10.56	< 0.001	[3.38, 4.92]
Impostor	0.56	0.07	0.25*	8.52	< 0.001	[0.43, 0.69]
Self-compassion	−1.02	0.08	−0.39*	−13.44	< 0.001	[−1.17, −0.88]

*p ≤ 0.001.

The third hierarchical regression (Table 4) examined whether self-compassion explained variance in depression beyond that of IP. In Step 1, IP was significantly associated with depression, *R*^2^ = 0.151, *F*(1, 1,206) = 215.18, *p* < 0.001, explaining 15.1% of the variance. Higher levels of IP were associated with increased depression (*β* = 0.389, *p* < 0.001). Step 2, which included self-compassion, significantly strengthened the model, *R*^2^ = 0.232, *F*(2, 1,205) = 181.58, *p* < 0.001. This model accounted for 23.2% of the variance in depression, representing a significant increase of 8.0%, Δ*R*^2^ = 0.080, *F*(1, 1,205) = 125.73, *p* < 0.001. With self-compassion in the model, the relationship between IP and depression remained significant but was meaningfully lower (*β* = 0.193, *p* < 0.001), and self-compassion demonstrated a strong negative association with depression (*β* = −0.344, *p* < 0.001). The effect size for self-compassion was large (*f*^2^ = 0.26).

**Table 4:** Examining variance in depression explained by IP and self-compassion.

	*B*	*SE*	*β*	*t*	*p*	95% CI
Step 1						
Constant	−0.87	0.21		−4.19	<0.001	[−1.28, −0.47]
Impostor	0.87	0.06	0.39*	14.67	<0.001	[0.75, 0.98]
Step 2						
Constant	3.10	0.41		7.63	<0.001	[2.30, 3.91]
Impostor	0.43	0.07	0.19*	6.30	<0.001	[0.30, 0.56]
Self-compassion	−0.88	0.08	−0.34*	−11.21	<0.001	[−1.03, −0.74]

*p ≤ 0.001.

### MANOVA analysis

MANOVA determined if anxiety, depression, and loneliness vary across levels of IP. IP scores were divided using sample specific tertile splits: Low IP (*n* = 456, *M* = 2.51, *SD* = 0.488), Moderate IP (*n* = 396, *M* = 3.56, *SD* = 0.226) and High IP (*n* = 373, *M* = 4.40, *SD* = 0.310).

**Figure 1:**
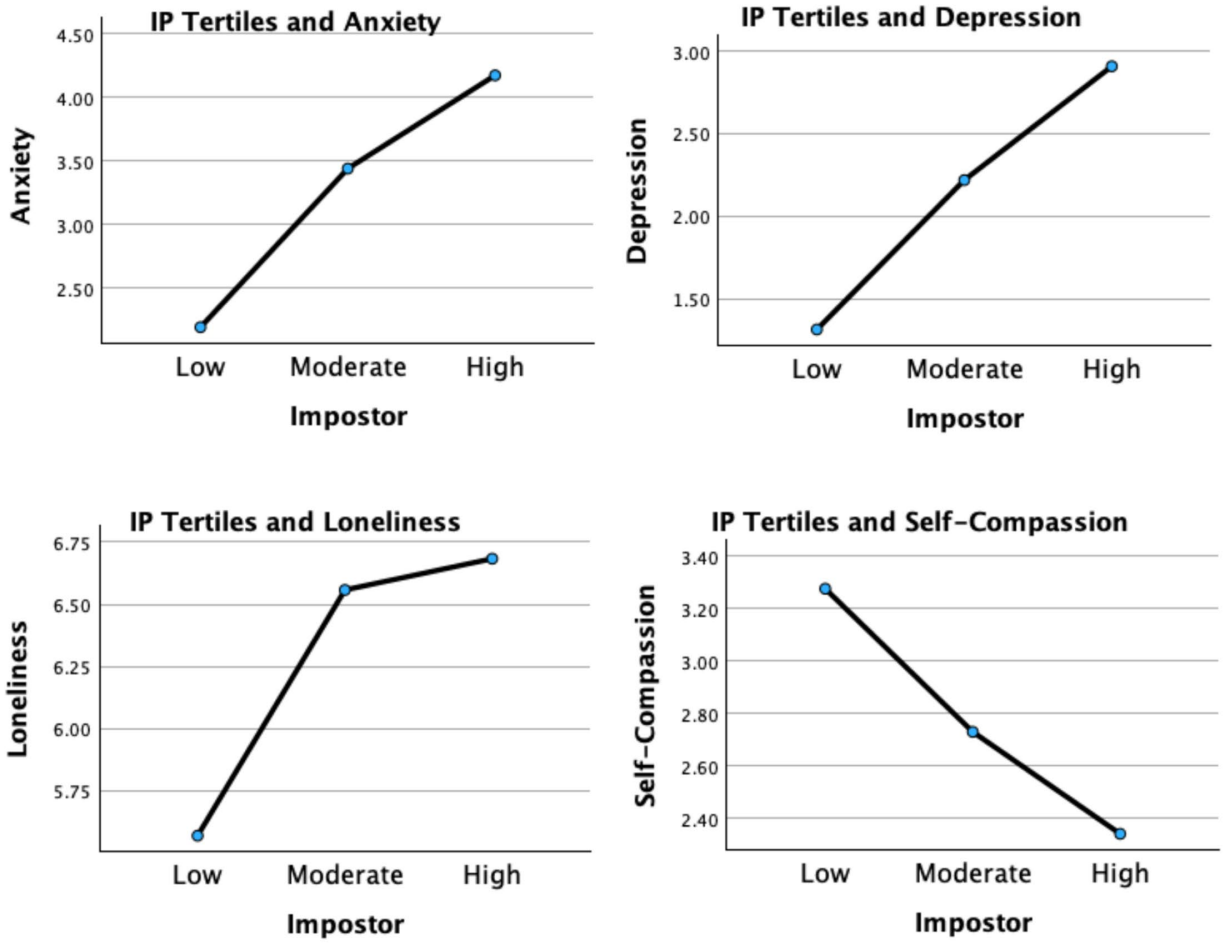
Self-compassion, depression, loneliness, and anxiety levels relative to IP tertiles.

Mental distress scores (anxiety, depression, and loneliness) varied significantly based on IP tertile with a large overall effect size, *F*(2, 1,220) = 60.765, *p* < 0.001; Wilk’s *Λ* = 0.692, *η*_p_^2^ = 0.168 (Figure 1). Anxiety scores increased in a linear fashion at each level of IP. Anxiety scores increased when IP rose from low to moderate with a medium effect (*d* = −0.694, *p* < 0.001), from moderate high with a small effect (*d* = −0.414, *p* < 0.001). Depression scores also differed significantly according to level of IP. Depression scores rose significantly as IP rose from low to moderate with a medium effect (*d* = −0.531, *p* < 0.001), from moderate to high with a small effect (*d* = −0.361, *p* < 0.001). Loneliness scores varied significantly as IP levels increased from low to moderate with a moderate effect (*d* = −0.530, *p* < 0.001), and from moderate to high (*d* = −0.072, *p* = 0.320). Loneliness was elevated at the low IP level and increased significantly at each IP level; however, there was no statistical difference in loneliness between moderate and high IP.

In contrast, IP had an inverse relationship pattern with self-compassion scores which decreased significantly as IP increased. Self-compassion diminished as IP rose from low to moderate with a large effect (*d* = 0.821, *p* < 0.001), and from moderate to high with a moderate effect size (*d* = 0.645, *p* < 0.001).

Anxiety, depression, and loneliness each increased most significantly as IP increased from the low to moderate level. These results support the hypothesis that even low and moderate levels of IP were significantly associated with poorer mental health and lower self-compassion.

## Discussion

### Impostor phenomenon among doctoral students

The findings suggest that a self-compassion framework may be a meaningful approach for mitigating impostor feelings and associated mental distress among doctoral students. The overall mean for IP (*M* = 3.43) among the present sample was skewed to the higher end of the IP range which was consistent with other doctoral student samples (Sverdlik et al., 2020; Tigranyan et al., 2021). This suggests that IP is common among doctoral students which may contribute to the graduate student mental health crisis (Evans et al., 2018).

Compared to male participants, female or gender non-binary students reported higher IP (Hutchins, 2015; Rackley et al., 2024). Impostor feelings do not happen in a vacuum, and it is possible that societal gender roles influenced female and non-binary students to feel a lack of belonging in doctoral education (Patzak et al., 2017; Rackley et al., 2024). In addition, our findings indicated an age effect with lower levels of IP reported by participants aged 40 or older, suggesting that students returning for a doctoral degree later in life may have less IP. This may be due to the development of effective coping skills or experienced greater emotional stability across their lived experience; however, more IP research can clarify the role of age and gender (Roberts et al., 2006).

The IP scores did not differ by race, area of study, or matriculation (Cokley et al., 2024). This non-significant difference is noteworthy and supports the universality of IP among graduate students across disciplines and cultures (Bravata et al., 2020; Clarke et al., 2024). While IP is prevalent among racial and ethnic minority groups, differing prevalence across cultures is not well supported in the literature (Cokley et al., 2024). A potential explanation is that common IP measures, such as the CIPS-10 used in this study, do not capture salient sociocultural factors or the intersectionality of identities that could more clearly assess IP in the context of racial or ethnic differences.

Furthermore, the lack of a statistically significant difference based on the year of matriculation is noteworthy. This may indicate internal characteristics such as personality traits or attributional styles may play a role in IP in addition to contextual factors (Brauer and Proyer, 2022). To our knowledge, no studies have compared IP rates across various academic disciplines, and it is possible that doctoral students may rate similarly high levels of IP across fields. However, the sources of IP may differ (e.g., research, teaching, applied practice). In addition, no statistically significant difference in the year of matriculation would suggest that IP is persistent throughout doctoral matriculation.

### Relationship between impostor phenomenon and mental distress

The results of the present study offer additional evidence of the strong relationship between IP and mental distress among doctoral students (Jackman et al., 2022; Pervez et al., 2020). In the present study, 702 (57.31%) participants exceeded the standard cutoff for moderate-to-severe depression, and 414 (33.80%) participants exceeded the cutoff for moderate-to-severe anxiety (Kroenke et al., 2009). While there is no particular cutoff score, the mean loneliness score is consistent with a recent doctoral study (Anderson, 2017).

Furthermore, IP correlated strongly with anxiety, depression, and loneliness (Table 1), which is consistent with previous research (e.g., Sverdlik et al., 2020; Tigranyan et al., 2021). The overall connection between IP and mental distress is not surprising, as feelings of self-doubt and the isolating fear of being discovered as an intellectual fraud are core components of IP (Bravata et al., 2020; Liu et al., 2023). More research on the relationship between IP and various mental distress variables is needed in order to better understand the full impact of IP on the well-being of doctoral students. With that said, our findings add to evidence of the relationships between IP and anxiety, depression, and loneliness (Jackman et al., 2022; Pervez et al., 2020).

In terms of demographic comparisons, there was no difference in anxiety, depression, and loneliness scores across ethnicity/race, age, discipline, or matriculation. However, findings suggested that female and non-binary doctoral students reported higher levels of anxiety than those who identified as male. Female and non-binary students may also face additional pressures academically, have less representation in academia, and potentially be more at risk for IP (Mills et al., 2024).

MANOVA analysis further explored the relationship between levels of IP and the mental distress variables (Table 4). This indicates that each increasing level of IP was related to significantly higher anxiety scores. The greatest increase in anxiety occurred as IP rose from low to moderate. Depression scores also increased linearly as the level of IP increased. The most significant increase in depression was noted as IP went from low to moderate, indicating that even lower levels of IP are associated with elevated depression among doctoral students. Loneliness scores in the sample increased as the IP level rose (Figure 1). This suggests that experiencing IP may increase loneliness in doctoral students, increasing the risk for mental distress and dropout (Janta et al., 2014). In contrast to distress, self-compassion scores decreased as IP increased, with the most significant reduction as IP rose from low to moderate. Overall, these results suggest IP is associated with mental distress even at lower levels and with lower self-compassion.

### The role of self-compassion and IP on mental distress

The mean score for self-compassion in our sample fell in the low-moderate range, which is slightly lower than recent doctoral student samples (Richardson et al., 2020; Solms et al., 2024; Tigranyan et al., 2021). With its high expectations, the competitive nature of doctoral training may challenge the self-kindness and acceptance central to self-compassion. Low self-compassion could also be related to confounding factors outside of our study design, such as limited exposure to contemplative practices or higher levels of mental distress specific to this doctoral sample. Interestingly, there were no significant differences in self-compassion based on demographics in the sample. In this study, higher levels of self-compassion were related to lower IP and mental distress.

Across all three regression models, self-compassion explained substantial additional variance beyond IP alone, with self-compassion consistently demonstrating strong negative associations with mental distress outcomes. The reduction in IP’s standardized coefficients when self-compassion was added to each model ranged from 47% (depression) to 75% (loneliness), suggesting it may represent an important correlate of mental health in the context of IP.

#### Self-compassion and impostor phenomenon in relation to loneliness

IP was related to higher loneliness in the sample, however, when considering.

self-compassion, IP was no longer statistically associated with loneliness. This suggests that self-compassionate students may experience IP with less isolation and loneliness. Doctoral education can be an isolating experience, especially during the dissertation phase, when students may have limited interaction with peers or support outside of their advisor. IP is strongly linked to loneliness and isolation, which often leads to mental distress, burnout, and poor academic outcomes (Blondeau, 2024; Ojeda, 2024). However, through normalizing struggle, self-compassionate doctoral students may be more likely to seek support and foster a sense of connectedness with peers and faculty (Neff, 2023; Posselt, 2018). Therefore, self-compassion’s emphasis on common humanity may be particularly important in reducing loneliness (Liu et al., 2023).

#### Self-compassion and impostor phenomenon in relation to anxiety

Self-compassion was associated with lower anxiety, accounting for 38.8% of the variance when considering IP in the model. While IP’s relationship with anxiety remained significant, it was reduced by nearly half. This is a meaningful finding given the high levels of anxiety among doctoral students and the close relationship between IP and anxiety both in this sample and in the broader literature (Pervez et al., 2020). Self-compassion, which is associated with lower anxiety, may help relieve distress while disrupting the IP cycle (Rosenscruggs and Schram, 2024).

#### Self-compassion and impostor phenomenon in relation to depression

In the final regression model, self-compassion accounted for 34.4% of the variance in depression and weakened the relationship between IP and depression. Inaccurately negative self-perceptions are a hallmark of IP, which can lead to depression, particularly when combined with the underlying anxiety common to IP (Gadsby and Hohwy, 2024). This finding adds evidence that self-compassionate students experiencing IP may report lower levels of depression (Clarke, 2024). Given the high prevalence of both IP and depression among doctoral students, this finding is crucial for both students and doctoral educators (Tigranyan et al., 2021).

### Benefits of self-compassion

Consistent with our theoretical framework, each of the regression models in this study were statistically significant, and the effect sizes suggest that the results are also practically meaningful. Overall, self-compassion was related to significantly lower IP, loneliness, anxiety, and depression among doctoral students. Overall, our results suggest that self-compassion is a promising avenue to help students navigate the emotional challenges of doctoral education despite experiencing IP.

Self-compassion is rooted in non-judgmental awareness, practicing self-kindness, and normalizing setbacks, all of which may help doctoral students manage self-doubt and inadequacy in a way that fosters a growth mindset rather than isolating self-criticism (Neff, 2023; Richardson et al., 2020). The doctoral journey is challenging; practicing self-acceptance and fostering a compassionate inner dialog may reduce distress and encourage students to build a supportive community and stronger academic collaborations. Doctoral students who approach challenges with self-compassion may have less fear of failure due to seeing inevitable setbacks as opportunities for growth rather than reflections of their personal inadequacy (Neff et al., 2005). This mindset can promote self-efficacy and persistence in the face of academic challenges (Clarke et al., 2024). By reducing the detrimental effects of perfectionism or procrastination, self-compassion may motivate students to take healthy risks, seek feedback, and engage more fully with their academic work, potentially leading to improved development (Richardson et al., 2020).

### Implications

The results of this study reveal that the prevalence and intensity of IP and related mental distress among doctoral students is a significant concern (Mills et al., 2024; Pervez et al., 2020; Tigranyan et al., 2021). A particularly concerning finding is that even lower levels of IP were related to significant anxiety, depression, and loneliness. The highly competitive and stressful environment in higher education can contribute to the prevalence and intensity of IP (Hutchins, 2015). Doctoral students often face pressure to publish and present scholarly work and to secure funding, all of which can foster unrealistic expectations and perfectionistic tendencies. This context renders doctoral students particularly vulnerable to IP, which increases the challenges in navigating academia and creates barriers to addressing IP-related mental distress (Bano and O'Shea, 2023; Pervez et al., 2020).

While IP and mental distress are ongoing concerns among doctoral students, our results indicate that self-compassion is a protective factor that is associated with lower IP and mental distress. Because even lower levels of IP relate to increased mental distress and that IP often increases during graduate training, addressing IP early in graduate training may be particularly important (Mulholland et al., 2023; Rosenscruggs and Schram, 2024). An early approach to self-compassion interventions may allow students time to develop effective coping and IP management skills. Doctoral programs can avoid inadvertently increasing IP with an intentional focus on coping rather than implying there is a quick cure (Rosenscruggs and Schram, 2024). Through increasing self-compassion, it may be possible to mitigate IP while supporting doctoral student retention (Waight and Giordano, 2018).

Self-compassion is a promising way to manage loneliness and isolation common to the doctoral experience (Charles et al., 2022). Since students experiencing IP often self-isolate, it could be particularly beneficial to encourage self-compassion within supportive relationships with faculty and mentors (Posselt, 2018). In fact, self-compassion workshops that bring together students with their advisors and faculty might lessen the power differential and foster greater openness and transparency in these relationships (Posselt, 2018; Sverdlik et al., 2020).

In addition to student-facing interventions, IP can be addressed by infusing self-compassion into the framework of doctoral programs and coursework. This approach may necessitate providing faculty and administrators with self-compassion training and resources that they can apply in courses, advisement, and collaborations. Integrating self-compassion into coursework may reach students who might not otherwise have access to interventions (Waight and Giordano, 2018). Integrating self-compassion within courses would take much less time than full-scale intervention programs such as Mindful Self-Compassion (MSC) and can take the form of readings, reflections, brief practice, and discussion (Germer and Neff, 2013). A compassionate approach to teaching and leadership can provide a culture shift and greater wellbeing for both students and faculty (Dreisoerner et al., 2023).

It is also important to recognize that students have differing needs, which may require an approach tailored to specific populations or disciplines (Bravata et al., 2020). For example, the different environments among doctoral programs could significantly influence students’ experiences of IP. The results of this study, which indicate that IP and mental distress were higher among students who identified as women and gender non-binary, can provide insights into how universities and programs can facilitate wellbeing and retain diverse students (Waight and Giordano, 2018). These and other contextual factors contributing to IP may include a lack of diverse representation in academia, stereotype threat, and systemic racism within higher education (Rosenscruggs and Schram, 2024). Environmental and contextual factors influence the prevalence of IP in higher education, suggesting the need for systemic institution-level change.

### Limitations and future directions

This study provides meaningful insights into the role of self-compassion in mitigating IP and related mental distress among U. S. doctoral students; however, several limitations are noted. First, the cross-sectional design precludes any definitive conclusions regarding causality. Longitudinal or experimental research is needed to establish temporal sequencing and determine if self-compassion causally buffers against IP-related outcomes over time (Liu et al., 2023).

While this study focused on self-compassion, other salient protective factors, such as social belonging, resilience, and perceived support from peers and faculty, were not examined. These variables may interact with self-compassion or independently relate to lower IP and warrant further exploration (Charles et al., 2022; Clarke and Guida, 2025). Additionally, this study used a single factor approach to IP, limiting the depth of interpretation. Future research can examine IP from a bi-factor conceptualization that includes *discount, fear of failure* and *luck* subscales in addition to the general IP factor (Brauer and Proyer, 2025). This would allow for greater specificity and shed light on potential mechanisms within IP.

Although the sample was large and broadly reflective of U. S. doctoral demographics, it was composed predominantly of White, cisgender, and female participants. This demographic profile may limit the generalizability of findings to more diverse or international doctoral populations (Cokley et al., 2024; Clarke et al., 2024). Additional work should explore how cultural background, and contextual and systemic influences shape experiences of IP and responses to self-compassion. Furthermore, participants who experienced heightened levels of IP or mental health concerns may have been more motivated to engage with the study, potentially introducing bias (Bano and O'Shea, 2023).

Finally, there is a need to evaluate the feasibility and effectiveness of self-compassion interventions tailored for doctoral students such as the MSC program (Germer and Neff, 2013). However, given limited time and high workload, scalable interventions, such as brief, structured practices or coursework-integrated modules, may be more accessible and sustainable (Solms et al., 2024; Waight and Giordano, 2018). In particular, Neff’s (2021) “fierce self-compassion” model, which emphasizes boundary-setting and empowered action, may hold unique value for women and individuals from marginalized backgrounds (Rackley et al., 2024).

## Conclusion

The doctoral journey is inherently challenging, requiring intellectual and emotional resilience. IP among doctoral students is a growing concern with ample evidence that mental health and wellbeing is dangerously compromised. This study underscores how IP exacerbates the severity of the mental health crisis, particularly due to the finding that even lower levels of IP are related to elevated anxiety, depression, and loneliness. Encouragingly, the present findings indicate that self-compassion may be a protective factor due to its association with positive mental health even in the presence of IP. Self-compassion may represent a framework associated with lower distress, bolstering their success.

## Data Availability

The raw data supporting the conclusions of this article will be made available by the authors, without undue reservation.
